# Help-seeking patterns for non-suicidal self-injury across borderline symptom profiles in a large sample of university students

**DOI:** 10.1186/s40479-026-00375-x

**Published:** 2026-09-25

**Authors:** Rosie James, Rebecca Overgaard, Sofie Westling, Magnus Nilsson

**Affiliations:** 1 Department of Clinical Sciences Malmo, Psychiatry, Lund University, Malmo, Sweden; 2 Department of Psychiatry, Skane University Hospital, Lund, Sweden

**Keywords:** Borderline personality disorder, Non-suicidal self-injury, Help-seeking, Internalizing, Externalizing, Latent class analysis, Social cognitive theory

## Abstract

**Background:**

Non-suicidal self-injury (NSSI) is common in young adults and may represent a marker of current or emerging borderline pathology. However, rates of help-seeking for NSSI are low, potentially delaying recognition of clinically relevant borderline symptoms. To our knowledge, research on NSSI help-seeking has not examined whether help-seeking differs according to underlying borderline symptom profiles. This study therefore examined whether patterns of NSSI help-seeking differ across borderline symptom profiles in a community sample of university students.

**Methods:**

In this cross-sectional study, latent class analysis was used to identify borderline symptom profiles in a community sample of 1,476 (68.5% female) Swedish university students based on the McLean Screening Instrument for BPD. Differences in help-seeking behavior, encouragement to seek help, help sources, and help-seeking attitudes were examined using three-step auxiliary analyses. Mixture logistic regression models assessed within-profile predictors.

**Results:**

Four borderline symptom profiles were identified in the full sample: Full Spectrum, Internalizing, Externalizing, and Asymptomatic. Among participants with lifetime NSSI (*n* = 581), help-seeking did not differ significantly across profiles, whereas encouragement to seek help and help-seeking attitudes did. Encouragement was the most consistent correlate of help-seeking across profiles, while attitudes were not reliably associated with help-seeking behavior. Among participants who had sought help and provided help-source data (*n* = 297), sources of help did not differ across profiles. There was no evidence that predictors of help-seeking differed across profiles.

**Conclusions:**

NSSI help-seeking did not differ significantly across borderline symptom profiles. Instead, encouragement from others to seek help was more consistently associated with help-seeking across profiles. This may be particularly important for individuals with more internalizing presentations, whose distress may be less visible and therefore less likely to elicit encouragement to seek support.

**Supplementary Information:**

The online version contains supplementary material available at https://doi.org/10.1186/s40479-026-00375-x.

## Background

Approximately one in eight young adults engage in non-suicidal self-injury (NSSI) worldwide [1], yet rates of help-seeking among those who self-injure remain low [2]. NSSI is the deliberate harm of one’s body tissue (e.g., cutting, burning, hitting) without the intention to die [3], and help-seeking can be understood as an adaptive coping process through which individuals seek external support [4]. Limited help-seeking is clinically important because NSSI is associated with anxiety and depression [5], increased risk of suicidal thoughts and behaviors [6], and borderline personality disorder (BPD) [7]. Self-injurious behavior is encompassed within one of the nine diagnostic criteria for BPD [8], is common among individuals with subthreshold borderline features [9, 10], and may emerge early in trajectories associated with later borderline pathology [11–13]. It may therefore represent an important marker of current or emerging borderline pathology, and help-seeking for NSSI may provide an opportunity for recognition of clinically relevant borderline symptoms and support needs.

However, borderline pathology is heterogeneous in its presentation. In addition to mixed presentations with broad symptom involvement, research has consistently identified patterns characterized by more internalizing or externalizing features [14–19]. Such heterogeneity may be particularly relevant in community populations, where subthreshold borderline symptoms can overlap with more common mental health complaints and may be less readily recognized [9, 20]. Symptom-level research further suggests that some internalizing borderline features, including emptiness, identity disturbance, and dissociation, are more strongly associated with NSSI than some externalizing features [21]. These differences raise the possibility that individuals who engage in NSSI may differ not only in their underlying borderline symptom configuration, but also in how their distress is recognized and how they enter help-seeking pathways. Borderline symptoms have previously been linked to delayed help-seeking among individuals who engage in NSSI [22], but it remains unclear whether this reflects borderline pathology more generally or varies according to symptom configuration. Whether NSSI help-seeking differs across borderline symptom profiles has not, to our knowledge, been examined.

Contemporary integrative frameworks, such as the Integrated Behavioral Model of Mental Health Help Seeking (IBM-HS), conceptualize help-seeking as a multistage process shaped by interacting individual, interpersonal, and structural determinants [23]. For the present study, two points in this process are particularly relevant: the transition into help-seeking and the subsequent pathway into care. Because disclosure may represent an important step toward obtaining interpersonal or professional support, Social Cognitive Theory (SCT) provides a useful lens for considering the intra- and interpersonal mechanisms involved and has recently been applied to NSSI disclosure [24]. In this work, disclosure is shaped by self-efficacy, outcome expectancies, and social modeling, with anticipated responses from others shaping expectations about social consequences and, in turn, influencing disclosure decisions. These findings suggest that interpersonal influences may function not only as contextual determinants, but as proximal social-cognitive processes that can facilitate or inhibit movement toward help-seeking. This provides a basis for considering whether differences in symptom presentation may alter the interpersonal context in which help-seeking occurs. In particular, borderline symptom profiles may be associated with differences in the visibility of distress and the responses elicited from others.

One possible mechanism is variation in the visibility and interpersonal expression of distress across symptom profiles [19], such that more externalizing features may elicit stronger interpersonal reactions, whereas internalizing features may be less detectable. If so, help-seeking may vary across profiles due to differences in how distress is expressed and recognized by others. Alternatively, help-seeking may be shaped less by symptom configuration and more by cross-cutting interpersonal processes, such as encouragement from others. Given the stigma associated with both NSSI and BPD [25, 26], individuals may delay help-seeking due to anticipated or experienced negative reactions, or because their distress is not recognized by others [27]. In this account, help-seeking may be relatively similar across symptom profiles, and depend on interpersonal input.

Taken together, these literatures support two testable accounts: a profile-specific account, in which symptom configuration shapes help-seeking behavior, and a profile-general account, in which help-seeking is driven more by cross-profile processes, such as encouragement from others to seek help. Clarifying this distinction has practical implications: if profile-specific differences exist, interventions may need to be tailored to presentation; if not, efforts may be better directed toward broadly enhancing interpersonal support and detection.

Accordingly, the present study examines whether NSSI help-seeking is better explained by profile-specific versus profile-general processes. Importantly, these profiles represent symptom-based configurations identified within a community sample and should not be interpreted as diagnostic BPD subtypes. Rather, they capture groups with differing patterns of borderline symptom endorsement, within which co-occurring NSSI may be clinically relevant to current or emerging borderline pathology. We asked the following research questions:

1. What borderline symptom profiles can be identified in this community sample of university students, and how do they differ on clinically relevant external variables?
2. Do help-seeking behaviors, help-seeking attitudes, encouragement to seek help, and source of help for NSSI differ across borderline symptom profiles among individuals who have engaged in NSSI?
3. Which factors predict help-seeking within each profile, and do these associations differ across profiles?

## Methods

### Design and procedure

We used a person-centered cross-sectional study design. Participants were eligible if they were enrolled students at the university at the time of recruitment. No clinical inclusion or exclusion criteria were applied. All enrolled students ($n = 32{,}001$) were invited via email to complete a comprehensive survey on self-harming behaviors and attitudes. Responses from 1,500 individuals were recorded and 24 individuals were removed based on a predefined attention check, in which participants were instructed to select a specific response option to verify that they were reading the survey questions. The full analytic sample (*N* = 1,476) was used to identify and validate the borderline symptom profiles. Analyses specifically concerning NSSI help-seeking, encouragement, and help-seeking attitudes were conducted among participants reporting lifetime NSSI (*n = 581*), as this was the population relevant to the study’s NSSI help-seeking questions. Analyses of help sources were further restricted to participants who reported help-seeking and had available help-source data (*n = 297*). Participation was voluntary and anonymous and no compensation was provided. All participants provided digital informed consent prior to taking the survey. The study was approved by the Swedish Ethical Review Authority (Dnr 2021–05102).

### Measures

#### Borderline personality disorder symptoms

McLean Screening Instrument for BPD (MSI-BPD [28]) was used to assess the number of borderline symptoms using 10 items with binary response options (yes/no). As two items represent one symptom according to the diagnostic procedure [8], this was condensed into a nine-item scale combining items 6 and 7 (paranoia/dissociation symptom) for analyses. Internal consistency in the present study was acceptable (Cronbach’s *α = 0.77*).

#### Help-seeking attitudes and behaviors

The Mental Help-Seeking Attitudes Scale (MHSAS [29]) was used to assess attitudes towards help-seeking. Mean scores were calculated from nine 7-point Likert scale answers (higher score = more positive). Internal consistency was high (Cronbach’s *α = 0.87*).

NSSI help-seeking behaviors were measured by asking those who reported engaging in NSSI at any point whether they had (1) ever sought help for NSSI, and (2) ever been encouraged to seek help for NSSI. Each question offered identical response options: “more than six months ago”, “within the last six months but not earlier”, “both within the last six months and earlier”, and “neither within the last six months nor earlier”. These were recoded into binary variables coded 0 (no, never) or 1 (yes, at some point). Those who had sought help were then also asked to indicate the source(s) of support, selecting from: “primary healthcare”, “psychiatry”, “student health”, “private healthcare”, “family and friends”, “internet/social media”, “community”, and “a helpline”. These were further collapsed into formal (first four) and informal (remaining) support categories for analysis.

#### NSSI and mental health measures

Non-suicidal self-injury (NSSI) was assessed with three survey items. The first asked whether participants had engaged in NSSI, with options reflecting timing: “more than six months ago”, “within the last six months but not earlier”, “both within the last six months and earlier”, and “neither within the last six months nor earlier”. Lifetime NSSI was defined as endorsement of any option except the last. The second and third items asked participants to indicate NSSI frequency within the last six months and more than six months ago, on a 7-point Likert scale between 0 (no times) and 6 (more than 5 times). For analyses, we created an operational repetition indicator coded 1 for * > 5* episodes in a given time window (*≤ 6* months; * > 6* months) and 0 otherwise. This is a frequency-based severity proxy.

Symptom Checklist-Core Depression Scale (SCL-CD6 [30]) was used to assess depressive symptoms using a 5-point Likert scale (higher score = more severe). Internal consistency was high (Cronbach’s *α = 0.90*).

Generalized Anxiety Disorder 7-item scale (GAD-7 [31]) was used to assess anxiety symptoms on a 4-point Likert scale (higher score = more severe). Internal consistency was high (Cronbach’s *α = 0.91*).

Self-Concept and Identity Measure (SCIM [32]) was used to measure identity pathology on a 7-point Likert scale, and includes three subscales: Consolidated Identity (e.g. “I know what I believe or value”), Disturbed Identity (e.g. “I change a lot depending on the situation”) and Lack of Identity (e.g. “I feel lost when I think about who I am”). For the total score, Consolidated Identity was reverse coded (higher total score = greater disturbance). It has been validated for use in its Swedish translation with a Cronbach’s *α* of 0.92 [33].

### Statistical analysis

We estimated a latent class analysis (LCA) on the full sample using nine MSI-BPD indicators (items 6–7 combined for the DSM-5 paranoia/dissociation criterion [8]) with robust maximum likelihood (MLR), extensive random starts, and replication of the best loglikelihood. Competing solutions (*k = 3*–*6*) were compared with standard information criteria (Akaike Information Criterion [AIC], Bayesian Information Criterion [BIC], adjusted BIC [aBIC]), likelihood ratio tests (Lo-Mendell-Rubin [LMR] and Bootstrap Likelihood Ratio Test [BLRT]) and substantive interpretability.

Between-profile differences were tested with the Bolck–Croon–Hagenaars (BCH) three-step method for continuous distal outcomes and the Distal Categorical (DCAT) three-step method for categorical distal outcomes. These are unadjusted comparisons that correct for classification error. Omnibus tests are primary; any pairwise tests are exploratory (unadjusted) and summarized in text/table notes. To examine within-profile predictors of help-seeking behavior, we fit one-step mixture logistic regression including encouragement, NSSI repetition (* > 5* episodes per window; *≤ 6* months vs * > 6* months), age, sex, and MHSAS as covariates. We allowed class-specific intercepts and slopes and tested their equality with a joint Wald test.

Class-indicator missingness was addressed via full-information maximum likelihood with robust standard errors (MLR). For BCH/DCAT auxiliary analyses (e.g., MHSAS, help-source, encouragement), Mplus applied listwise deletion; the sample size for each distal outcome is reported in the Results. Descriptive analyses were conducted in Stata/SE 19.5 [34] and latent class and mixture models (including BCH/DCAT analyses) were conducted in Mplus 8.11 [35].

This study is reported in line with recommendations for observational research reporting (STROBE guidelines).

## Results

### Participant characteristics

The analytic sample consisted of 1,476 students ($M_{\mathrm{age}} = 26.27$ years, *SD = 7.42*; 68.5% female). Among participants reporting lifetime NSSI (*n = 581*), descriptive comparisons between help-seekers and non-help-seekers are in Supplementary Table S1 (see Additional file 1).

### Latent class analysis

**Fig. 1:**
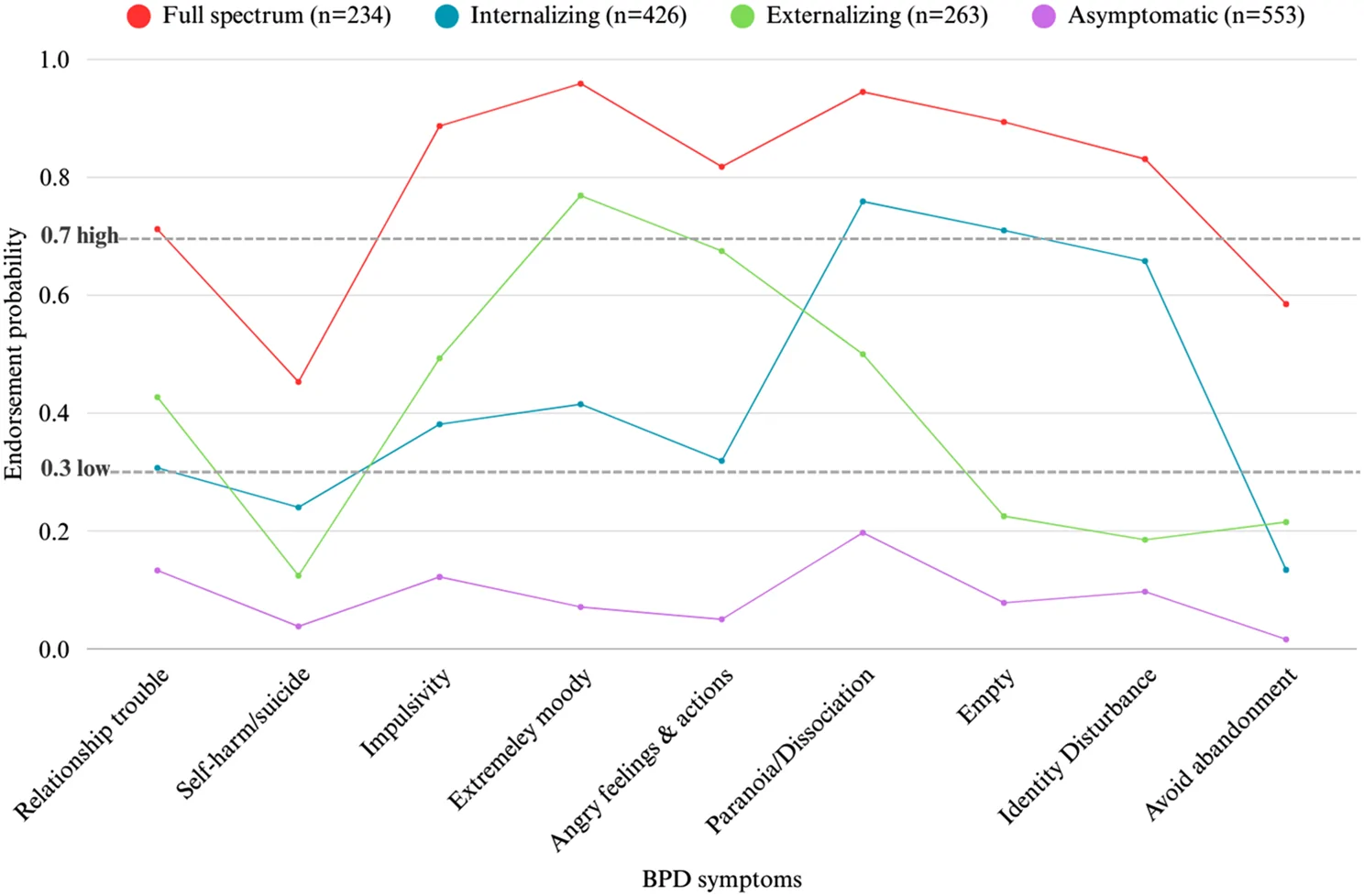
Borderline symptom profiles from the four-class latent class

The four-class solution showed the best balance of statistical fit and interpretability. Classification quality was adequate (entropy = 0.665; mean posterior probabilities: Full Spectrum = 0.82, Internalizing = 0.76, Externalizing = 0.74, Asymptomatic = 0.90). Model-selection indices are reported in Supplementary Table S2 (see Additional file 1). Figure 1 shows the estimated item-endorsement probabilities for the nine MSI-BPD indicators, with * > 0.7* representing high endorsement and * < 0.3* representing low endorsement [36]. These classes represent patterns of borderline symptom endorsement within the community student sample and do not correspond to clinical diagnoses or diagnostic subtypes.

Class 1 (Full Spectrum, *n = 234*; 15.8%) showed high endorsement of most BPD symptoms. Class 2 (Internalizing, *n = 426*; 28.9%) showed high emptiness, identity disturbance and paranoia/dissociation symptoms. Class 3 (Externalizing, *n = 263*; 17.8%) showed elevated endorsement of mood instability and anger. Class 4 (Asymptomatic, *n = 553*; 37.5%) showed low endorsement of all BPD symptoms. Overall sample characteristics are presented in the Total column of Table 1.

**Table 1:** Descriptive characteristics of the four borderline symptom profiles

	*Borderline symptom profile*				
	Full Spectrum	Internalizing	Externalizing	Asymptomatic	Total
N	234 (15.85%)	426 (28.86%)	263 (17.82%)	553 (37.47%)	1476 (100%)
Age	25.38 (5.30)	25.01 (5.66)	26.79 (7.33)	27.37 (9.07)	26.27 (7.42)
*Female*					
Yes	175 (74.79%)	283 (66.43%)	206 (78.33%)	347 (62.75%)	1011 (68.50%)
*Lives with others*					
Yes	130 (56.77%)	234 (56.93%)	176 (69.29%)	371 (68.32%)	911 (63.40%)
*In a relationship*					
Yes	111 (47.44%)	150 (35.21%)	160 (60.84%)	270 (48.82%)	691 (46.82%)
Lifetime NSSI	163 (69.66%)	195 (45.77%)	95 (36.12%)	128 (23.15%)	581 (39.4%)
*Repetitive NSSI*					
No repetitive	41 (25.15%)	57 (29.23%)	30 (31.58%)	52 (40.62%)	180 (30.98%)
Recent only	2 (1.23%)	5 (2.56%)	0 (0.00%)	0 (0.00%)	7 (1.20%)
Prior only	96 (58.90%)	111 (56.92%)	58 (61.05%)	72 (56.25%)	337 (58.00%)
Both	24 (14.72%)	22 (11.28%)	7 (7.37%)	4 (3.12%)	57 (9.81%)

*Note*. Values are means (SD) or n (%). Repetitive NSSI categories indicate self-injury occurring more than five times (recent = within 6 months; prior = more than 6 months). Percentages are based on modal class assignment and do not account for classification uncertainty; therefore, subgroup counts may differ in subsequent analyses

### Validation of the borderline symptom profiles

Using BCH three-step models, all clinical validators differed significantly across profiles (*p < .001*). As shown in Table 2, the Full Spectrum profile showed the highest depression, anxiety, identity pathology, and emotion dysregulation, whereas the Asymptomatic profile showed the lowest levels. Internalizing and Externalizing profiles fell between these extremes and did not differ significantly in levels of anxiety.

**Table 2:** Profile differences in depression, anxiety, identity pathology, and emotion dysregulation (BCH; *N = 1476*)

	Full Spectrum	Internalizing	Externalizing	Asymptomatic	Statistic
SCL-CD6	23.13 (0.48)$^{a}$	19.24 (0.41)$^{b}$	15.21 (0.49)$^{c}$	11.11 (0.24)$^{d}$	$\chi^2(3)=720.50$, *p < .001*
GAD-7	20.58 (0.45)$^{a}$	16.13 (0.37)$^{b}$	15.28 (0.44)$^{b}$	10.70 (0.19)$^{c}$	$\chi^2(3)=583.54$, *p < .001*
SCIM	100.44 (2.12)$^{a}$	88.51 (1.47)$^{b}$	64.02 (1.70)$^{c}$	57.18 (0.83)$^{d}$	$\chi^2(3)=645.55$, *p < .001*
DERS-16	58.90 (1.04)$^{a}$	44.69 (0.84)$^{b}$	39.52 (1.04)$^{c}$	28.09 (0.48)$^{d}$	$\chi^2(3)=918.42$, *p < .001*

*Note*. Values are estimated means (SE) from BCH; *χ*^2^ tests are omnibus class-difference tests. Letters denote pairwise differences at *p* < .05 (unadjusted); profiles sharing a letter do not differ significantly. Profile counts are based on estimated posterior probabilities

### Help-seeking across borderline symptom profiles

Among participants with lifetime NSSI (*n = 581*), the omnibus DCAT test of profile differences in help-seeking behavior was non-significant ($\chi^2(3)=7.10$, *p=.069*). Exploratory pairwise contrasts indicated higher help-seeking in the Full Spectrum compared with the Asymptomatic profile (*p=.011*); other contrasts were non-significant. Model-estimated probabilities showed a modest gradient (Table 3).

**Table 3:** Help-seeking behavior and attitudes by borderline symptom profile (lifetime NSSI; *N = 581*)

	Full Spectrum	Internalizing	Externalizing	Asymptomatic
Help-seeking, probability (DCAT; SE)	0.63 (0.05)$^{a}$	0.50 (0.04)$^{ab}$	0.51 (0.08)$^{ab}$	0.45 (0.05)$^{b}$
Encouragement to seek help (DCAT; SE)	0.61 (0.05)$^{a}$	0.41 (0.06)$^{b}$	0.53 (0.10)$^{a}$	0.41 (0.05)$^{b}$
MHSAS (mean, SE)	5.08 (0.11)$^{a}$	5.27 (0.12)$^{ab}$	5.72 (0.19)$^{b}$	5.58 (0.12)$^{b}$

*Note*. Three-step BCH/DCAT adjusts for classification error. Omnibus: help-seeking *χ*^2^ (3) = 7.10, p = .069; MHSAS *χ*^2^ (3) = 14.87, *p* = .002; encouragement *χ*^2^ (3) = 10.55, *p* = .014. Letters denote exploratory pairwise differences at *p* < .05 (unadjusted)

Encouragement to seek help differed across profiles (three-step DCAT omnibus $\chi^2(3)=10.55$, *p=.014*). Exploratory pairwise contrasts indicated higher encouragement in the Full Spectrum relative to the Internalizing (*p=.010*) and Asymptomatic (*p=.003*) profiles; other contrasts were non-significant.

Help-seeking attitudes also differed across profiles among those with lifetime NSSI ($\chi^2(3)=14.87$, *p=.002*). The Full Spectrum profile reported the least positive attitudes (*M = 5.08*), significantly lower than the Externalizing (*M = 5.72*) and Asymptomatic (*M = 5.58*) profiles (Table 3). Differences among the remaining profiles were small and non-significant.

### Sources of help-seeking

**Fig. 2:**
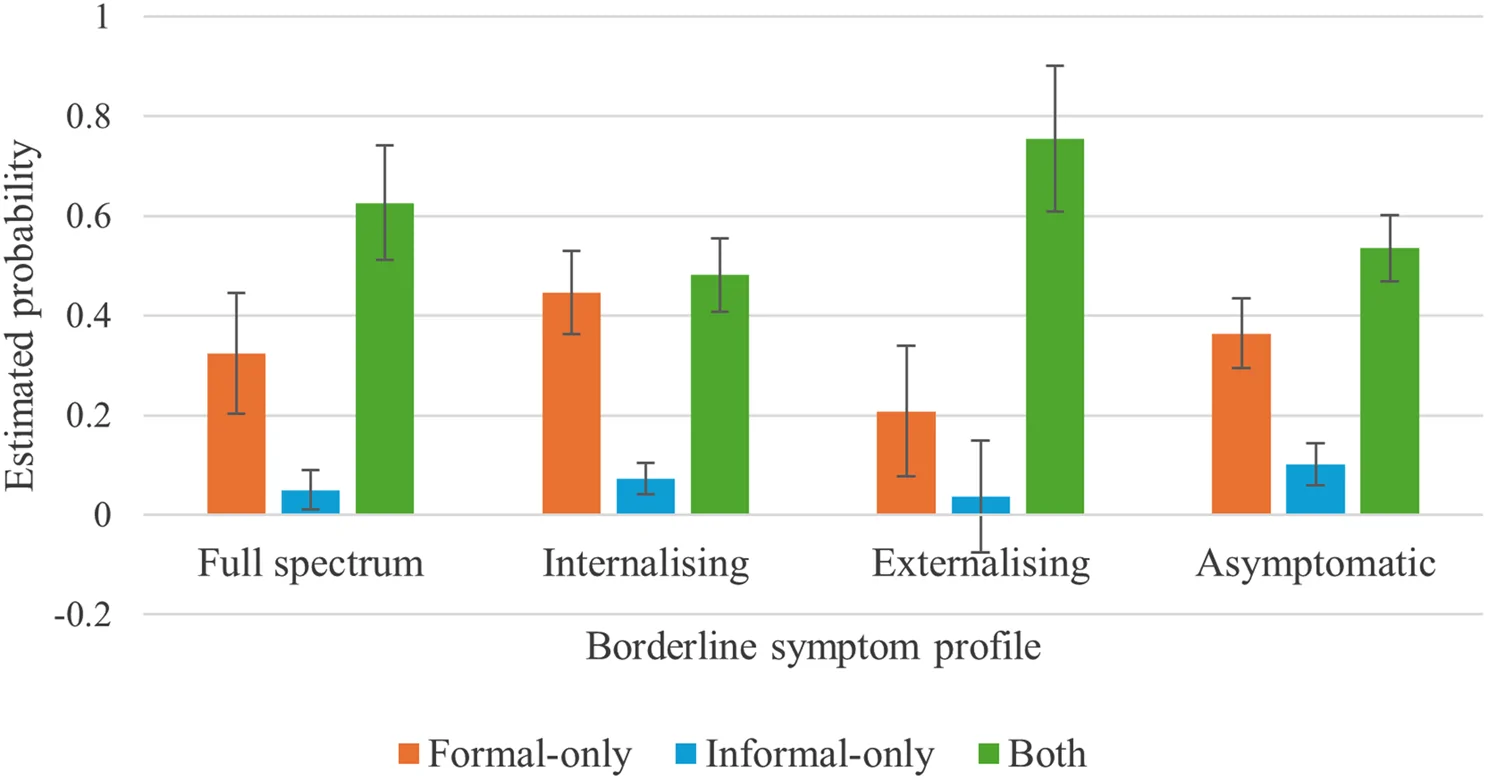
Estimated probabilities of help source category by borderline symptom profile conditional on help-seeking (*N = 297*)

Of 581 students with lifetime NSSI, 304 (52.3%) reported any help-seeking; help source data were available for 297 due to missing data on one or more help-seeking variables (handled via listwise deletion). Profile-specific denominators and category counts are reported in Supplementary Table S3 (see Additional file 1). Figure 2 shows the estimated probabilities of help source categories by borderline symptom profile. Help source categories did not differ significantly by profile ($\chi^2(6)=7.74$, *p=.258*).

### Predictors of help-seeking within profiles (one-step mixture regression; MLR)

One-step mixture logistic regressions were conducted (outcome = ever sought help) within each profile, adjusting for age, sex, NSSI repetition/recency, encouragement, and MHSAS (Table 4). Encouragement to seek help was associated with higher odds of seeking help in all profiles, reaching statistical reliability in the Full Spectrum (OR = 17.87; 95% CI [5.27, 60.57]) and Asymptomatic (OR = 3.31; 95% CI [1.20, 9.12]) profiles; estimates were positive but imprecise for the remaining profiles.

**Table 4:** Ors (95% CIs) from a one-step mixture regression predicting NSSI help-seeking (*N = 581*)

	Full Spectrum	Internalizing	Externalizing	Asymptomatic
Age	1.10 (0.94, 1.28)	0.97 (0.90, 1.05)	0.98 (0.85, 1.12)	0.96 (0.87, 1.07)
Female	1.20 (0.35, 4.12)	1.28 (0.40, 4.06)	0.17 (0.01, 3.62)	1.43 (0.42, 4.87)
Recent NSSI	2.90 (0.33, 25.59)	1.04 (0.35, 3.10)	0.92 (0.02, 39.74)	0.14 (0.01, 1.48)
Past NSSI	0.76 (0.18, 3.11)	3.26 (1.32, 8.07)$^{*}$	2.62 (0.23, 30.50)	3.12 (1.19, 8.19)$^{*}$
Encouragement	17.87 (5.27, 60.57)$^{*}$	1.97 (0.79, 4.94)	3.77 (0.34, 41.80)	3.31 (1.20, 9.12)$^{*}$
MHSAS	0.92 (0.50, 1.68)	1.16 (0.76, 1.76)	1.86 (0.65, 5.32)	1.10 (0.73, 1.66)

*Note*. ORs (95% CIs) from a one-step mixture logistic regression; outcome = ever help-seeking (N = 581). ^*^95% CI excludes 1. Predictor prevalence is reported in Supplementary Table S4.

Repetitive NSSI more than 6 months ago predicted help-seeking in the Internalizing (OR = 3.26, 95% CI [1.32, 8.07]) and Asymptomatic (OR = 3.12, 95% CI [1.19, 8.19]) profiles; associations were not reliable for the other profiles. Recent repetition, attitudes, age, and sex showed no reliable associations in any profile.

The adjusted one-step mixture preserved the qualitative ordering of help-seeking across profiles (highest in Full Spectrum, lowest in Asymptomatic) and showed no evidence of cross-profile heterogeneity: a joint Wald test of all class-specific thresholds and slopes was non-significant ($\chi^2(21)=20.11$, *p=.514*).

## Discussion

We examined whether self-reported history of help-seeking for NSSI is better explained by a profile-specific account, in which help-seeking varies across borderline symptom profiles, or by a profile-general account, in which help-seeking is shaped more by processes operating across profiles. The identified borderline symptom profiles (Full Spectrum, Internalizing, Externalizing, Asymptomatic) were consistent with commonly identified underlying dimensions of BPD in clinical and community samples [19].

Importantly, the aim of the latent class analysis was not to identify groups meeting diagnostic thresholds for BPD, but to examine whether meaningful configurations of borderline symptoms could be distinguished within a community population. Whereas the Asymptomatic and Full Spectrum profiles broadly reflected lower and higher overall levels of symptom endorsement, the Internalizing and Externalizing profiles differed qualitatively in their patterns of symptom endorsement rather than simply reflecting increasing overall levels of symptoms. Thus, the identified profiles should be interpreted as symptom configurations rather than diagnostic or subthreshold BPD groups. The relatively low mean level of borderline symptoms in the overall sample is consistent with its community-based nature, while remaining compatible with the identification of clinically relevant variation in symptom presentation. Evidence suggests that even subthreshold borderline features may be associated with clinically meaningful impairment and risk [9].

While these profiles differed meaningfully from each other in terms of help-seeking attitudes and indicators of psychological distress, these differences did not translate into robust variation in help-seeking behavior. Instead, help-seeking was more consistently associated with encouragement from others to seek help.

### Help-seeking differences and similarities across borderline symptom profiles

The identified profiles showed expected differences in measures of psychopathology, with the Full Spectrum and Internalizing profiles characterized by higher levels of depression, emotional dysregulation, and identity disturbance, and descriptively greater repetitive NSSI engagement than the Externalizing and Asymptomatic profiles. These findings align with prior work indicating that internalizing presentations are characterized by greater affective distress and self-harm, whereas externalizing presentations are marked by impulsivity and outwardly expressed dysregulation, such as intense anger, interpersonal conflict, and impulsive actions [19]. This further supports the relevance of internalizing–externalizing distinctions within borderline pathology, even in community samples [14, 37].

We interpret these findings in relation to two stages of the help-seeking process: first, the transition into help-seeking (i.e., recognition of NSSI and the decision to seek support), and second, the route into care (i.e., whether support is accessed through formal or informal sources).

At the first stage, we identified differences across profiles in the likelihood of being encouraged to seek help for NSSI. Individuals in the Full Spectrum profile were more often encouraged than those in the Internalizing and Asymptomatic profiles, but not the Externalizing profile. Given that the Internalizing profile reports rates of repetitive NSSI comparable to the Full Spectrum profile, this difference is unlikely to be explained solely by NSSI frequency.

One interpretation is that this reflects differences in the visibility or disclosure of distress. Presentations characterized by more outwardly expressed dysregulation, such as interpersonal conflict and impulsive actions [38], may make distress more observable to others, whereas more internalizing presentations may involve greater concealment of distress [39] and less opportunity to receive encouragement from others to seek support.

Help-seeking attitudes also differed across profiles. The Full Spectrum profile reported less positive attitudes toward help-seeking than the Externalizing and Asymptomatic profiles, while the remaining pairwise differences were not significant. The less positive attitudes observed in the Full Spectrum profile may partly reflect the greater stigma burden associated with borderline pathology, including anticipated negative reactions and internalized stigma [26, 40, 41]. They may also reflect the combined burden of co-occurring internalizing and externalizing symptoms, consistent with evidence that borderline pathology lies at the intersection of these dimensions of psychopathology [42, 43]. A similar interpretation may apply to the Internalizing profile, given its association with heightened internal distress and self-critical cognitions [44], although evidence distinguishing internalizing from externalizing presentations in help-seeking attitudes remains limited.

Of the two help-seeking constructs, encouragement, but not attitudes, predicted help-seeking behavior. This mismatch between attitudes and behavior is commonly observed in help-seeking research [4, 45, 46]. Our findings highlight the importance of interpersonal encouragement in the transition from distress to help-seeking. The pattern is consistent with profile differences in encouragement contributing to differences in help-seeking. Interpersonal encouragement was more consistently associated with help-seeking than help-seeking attitudes.

At the second stage, we found no clear differences across profiles in whether support was accessed through formal or informal routes. Once help-seeking was initiated, pathways into care were broadly similar across profiles. The most common response was the use of both formal and informal support, rather than a clear preference for one source. This contrasts with findings suggesting a preference for informal sources [25], and instead indicates that help-seeking for NSSI commonly involves engagement with formal care, often alongside informal support. Help-seeking pathways therefore appeared to converge across profiles [45, 47].

### Implications for help-seeking theory

Our results point to the early stages of help-seeking as particularly important, when distress must be recognized and acted upon. Encouragement emerged as a particularly important interpersonal factor in movement toward help-seeking.

Encouragement from others may function as a bridge in the help-seeking pathway by validating distress, reducing perceived stigma, and increasing confidence in seeking support. From a Social Cognitive Theory perspective, encouragement can be conceptualized as a form of social influence that shapes outcome expectancies, enhances self-efficacy, and provides behavioral modeling [24]. This interpretation aligns with integrative frameworks such as the Integrated Behavioral Model of Mental Health Help-Seeking [23], which conceptualize help-seeking as arising from interacting individual, interpersonal, and structural determinants.

Help-seeking was more consistently associated with encouragement than with attitudes or symptom configuration. This highlights the potential value of validation, acknowledgment, and active encouragement from both informal supports and care providers.

### Implications for timely recognition of borderline symptoms

As help-seeking for NSSI may provide an opportunity to identify clinically relevant borderline symptoms even when they remain below diagnostic thresholds, processes that facilitate help-seeking may also support more timely identification. While the help-seeking pathway appears broadly similar once engagement occurs, access to this pathway may depend on interpersonal detection. In the present context, timely identification refers to recognition of clinically relevant symptoms and support needs at subthreshold levels, rather than detection close to the initial onset of NSSI.

Encouragement from others was more consistently associated with help-seeking than differences between borderline symptom profiles, suggesting that interpersonal influences warrant greater attention in efforts to support recognition and service engagement. This may be particularly important for individuals with internalizing presentations, whose distress may be less visible and therefore less likely to prompt encouragement from others.

Although help-seeking behavior did not differ significantly across profiles, the lower likelihood of receiving encouragement among individuals with Internalizing presentations may represent one route to delayed access to support.

These findings do not imply that co-occurring NSSI and borderline symptoms necessarily indicate a need for acute intervention. Rather, they highlight opportunities for recognition and assessment of support needs, for example through normalizing conversations about NSSI and encouraging help-seeking [47]. Such approaches may be particularly relevant for individuals in early points of contact, such as primary care or university support services, where uncertainty about responding to self-harm is common [48]. Universities may therefore offer valuable opportunities for low-threshold, interpersonal support pathways, particularly in contexts where NSSI prevalence is high [49].

### Strengths and limitations

A large student sample allowed for person-centered modeling of borderline heterogeneity in a community context, shifting focus from diagnostic thresholds to meaningful subthreshold patterns. This approach aligns well with clinical practice [50]. The combined use of three-step (BCH/DCAT) and one-step mixture approaches enabled the integration of between-profile comparisons with within-profile prediction while accounting for classification error.

Several limitations should be noted. First, the response rate was low and participation was voluntary, introducing the possibility of selection bias. Participants who chose to respond may have differed systematically from non-responders, although the direction of such bias cannot be determined from our data. The sample should therefore not be considered representative of a defined prodromal or at-risk BPD population. Rather, the study examines variation in borderline symptom patterns within a self-selected, community sample of university students, and prevalence estimates of the identified profiles should be interpreted with caution.

Second, information on alcohol and substance use was not collected, limiting our ability to characterize externalizing features beyond NSSI. Future research should incorporate broader indicators such as substance use or conduct problems.

Third, the repetition indicator represents a frequency-based severity proxy and does not correspond to a standardized measure of NSSI. We did not assess NSSI methods or medical severity, limiting our ability to examine whether these characteristics differed across symptom profiles or were associated with help-seeking. Neither did we assess the age at NSSI onset nor the duration of NSSI. The category “more than six months ago” therefore indicates only that NSSI occurred at some point prior to the six-month assessment window and does not provide information about when NSSI began or how long it persisted. Consequently, we cannot determine how the timing or duration of NSSI may relate to participants’ current borderline symptom profiles.

Fourth, the cross-sectional design precludes conclusions about temporal ordering between encouragement and help-seeking. Furthermore, encouragement was assessed only in terms of whether and approximately when it had occurred. We did not assess its source, form, intensity, or content, and therefore cannot distinguish between brief verbal encouragement and more active or sustained support in accessing care. Future research should examine these encouragement–engagement mechanisms in greater detail. We also lacked information on previous or ongoing treatment, including psychotherapy, medication, treatment specifically targeting BPD, and treatment outcomes. Although help-seeking was assessed using broad temporal categories, the cross-sectional design does not allow us to establish the temporal relationship between help-seeking and current symptom levels. Consequently, we cannot determine whether previous help or treatment contributed to lower current levels of borderline symptoms or NSSI.

### Conclusion

NSSI help-seeking did not differ significantly across borderline symptom profiles, despite meaningful profile differences in psychological distress, help-seeking attitudes, and likelihood of receiving encouragement. Encouragement from others was the most consistent factor associated with help-seeking, while sources of help were broadly similar across profiles once help-seeking had occurred.

Overall, the findings point to interpersonal processes as particularly relevant to the initiation of NSSI help-seeking, consistent with multistage and social-cognitive accounts of help-seeking. As NSSI can occur alongside clinically relevant borderline symptoms and in the context of emerging borderline pathology, improving recognition of NSSI-related distress and encouraging help-seeking may create opportunities for timely identification of support needs. This may be especially important for individuals with more internalizing presentations, whose distress could be less visible to others and consequently less likely to elicit encouragement to seek support.

## Electronic supplementary material

Below is the link to the electronic supplementary material.

Supplementary Material 1: Additional file 1. *Supplementary information* (.docx). Contains analysis syntax and supplementary tables: S1, Descriptive comparisons of help seekers vs non help seekers (lifetime NSSI subgroup); S2, Latent class model criterion for model selection; S3, Help source denominators and counts by profile among help-seekers (*n* = 297); and S4, Prevalence of key binary predictors by borderline symptom profile among students with lifetime NSSI (*n* = 581).

## Data Availability

The datasets generated and/or analysed during the current study are not publicly available due to ethical restrictions related to participant confidentiality but are available from the corresponding author on reasonable request. The analysis code used in this study is available on the Open Science Framework at https://doi.org/10.17605/OSF.IO/APS3M.

## Abbreviations

BPD: Borderline Personality Disorder
NSSI: Non-Suicidal Self-Injury
LCA: Latent Class Analysis
BCH: Bolck–Croon–Hagenaars 3-step method
DCAT: Distal Categorical 3-step method

## References

[1] Swannell SV, Martin GE, Page A, Hasking P, John NJS. Prevalence of nonsuicidal self-injury in nonclinical samples: systematic review, meta-analysis and meta-regression. Suicide Life Threat Behav. 2014;44(3):273–303. 10.1111/sltb.12070. 24422986

[2] Barnett P, Tickell A, Osborn T, Delamain H, Fonagy P, Pilling S, et al. Help-seeking and disclosure in university students with suicidal thoughts and self-harm: a systematic review. Int J Ment Health Addict. 2024;24(1):623–700. 10.1007/s11469-024-01416-8.

[3] Nock MK, Prinstein MJ. A functional approach to the assessment of self-mutilative behavior. J Consult Clin Psych. 2004;72(5):885–90. 10.1037/0022-006X.72.5.885. 15482046

[4] Rickwood DT. Conceptual measurement framework for help-seeking for mental health problems. Phychol Res Behav Manag. 2012;12;p. 173. 10.2147/PRBM.S38707. PMC3520462 23248576

[5] Bentley KH, Cassiello-Robbins CF, Vittorio L, Sauer-Zavala S, Barlow DH. The association between nonsuicidal self-injury and the emotional disorders: a meta-analytic review. Clin Psychol Rev. 2015;37:72–88. 10.1016/j.cpr.2015.02.006. 25771494

[6] Kiekens G, Hasking P, Boyes M, Claes L, Mortier P, Auerbach RP, et al. The associations between non-suicidal self-injury and first onset suicidal thoughts and behaviors. J Affective Disord. 2018;239:171–79. 10.1016/j.jad.2018.06.033. 30014957

[7] Turner BJ, Dixon-Gordon KL, Austin SB, Rodriguez MA, Rosenthal MZ, Chapman AL. Non-suicidal self-injury with and without borderline personality disorder: differences in self-injury and diagnostic comorbidity. Psychiatry Res. 2015;230(1):28–35. 10.1016/j.psychres.2015.07.058. 26315664

[8] Association AP. Diagnostic and statistical manual of mental disorders. 5th. American Psychiatric Association Publishing; 2022.

[9] Thompson KN, Jackson H, Cavelti M, Betts J, McCutcheon L, Jovev M, et al. The clinical significance of subthreshold borderline personality disorder features in outpatient youth. J Pers Disord. 2019;33(1):71–81. 10.1521/pedi_2018_32_330. 30036169

[10] Brager-Larsen A, Zeiner P, Mehlum L. Sub-threshold or full-syndrome borderline personality disorder in adolescents with recurrent self-harm – distinctly or dimensionally different? Borderline Pers Disord Emot Dysregul. 2023;10(1):26. 10.1186/s40479-023-00234-z. PMC10500832 37705040

[11] Biskin RS, Paris J, Zelkowitz P, Mills D, Laporte L, Heath N. Nonsuicidal self-injury in early adolescence as a predictor of borderline personality disorder in early adulthood. J Pers Disord. 2021;35(5):764–75. 10.1521/pedi_2020_34_500. 33779286

[12] Reichl C, Kaess M. Self-harm in the context of borderline personality disorder. Curr Opin Psychol. 2021;37:139–44. 10.1016/j.copsyc.2020.12.007. 33548678

[13] Nakar O, Brunner R, Schilling O, Chanen A, Fischer G, Parzer P, et al. Developmental trajectories of self-injurious behavior, suicidal behavior and substance misuse and their association with adolescent borderline personality pathology. J Affective Disord. 2016;197:231–38. 10.1016/j.jad.2016.03.029. 26995466

[14] Gamache D, Savard C, Leclerc P, Payant M, Côté A, Faucher J, et al. Latent profiles of patients with borderline pathology based on the alternative DSM-5 model for personality disorders. Borderline Pers Disord Emot Dysregul. 2021;8(1):4. 10.1186/s40479-021-00146-w. PMC7876791 33568234

[15] Oladottir K, Wolf-Arehult M, Ramklint M, Isaksson M. Cluster analysis of personality traits in psychiatric patients with borderline personality disorder. Borderline Pers Disord Emot Dysregul. 2022;9(1):7. 10.1186/s40479-022-00178-w. PMC8822819 35130981

[16] Wolf K, Scharoba J, Noack R, Keller A, Weidner K. Subtypes of borderline personality disorder in a day-clinic setting—clinical and therapeutic differences. Personality Disord: Theory Res Treat. 2023;14(5):555–66. 10.1037/per0000624. 37561474

[17] Smits ML, Feenstra DJ, Bales DL, de Vos J, Lucas Z, Verheul R, et al. Subtypes of borderline personality disorder patients: a cluster-analytic approach. Borderline Pers Disord Emot Dysregul. 2017;4(1):16. 10.1186/s40479-017-0066-4. PMC5494904 28680639

[18] Ramos V, Canta G, de Castro F, Leal I. Discrete subgroups of adolescents diagnosed with borderline personality disorder: a latent class analysis of personality features. J Pers Disord. 2014;28(4):463–82. 10.1521/pedi_2013_27_126. 24344843

[19] Triantafyllou A, Konstantakopoulos G, Stefanatou P, Giannouli E, Malogiannis IA. Underlying dimensions of borderline personality disorder: a systematic review of factor analytic studies. Psychiatric Quart. 2025;96(4):751–85. 10.1007/s11126-025-10141-x. PMC12647336 40186846

[20] Köhne ACJ, Isvoranu AM. A network perspective on the comorbidity of personality disorders and mental disorders: an illustration of depression and borderline personality disorder. Front Phychol. 2021;12. 10.3389/fpsyg.2021.680805. PMC8290338 34295287

[21] Brickman LJ, Ammerman BA, Look AE, Berman ME, McCloskey MS. The relationship between non-suicidal self-injury and borderline personality disorder symptoms in a college sample. Borderline Pers Disord Emot Dysregul. 2014;1(1):14. 10.1186/2051-6673-1-14. PMC4579519 26401298

[22] Lustig S, Koenig J, Resch F, Kaess M. Help-seeking duration in adolescents with suicidal behavior and non-suicidal self-injury. J Psychiatric Res. 2021;140:60–67. 10.1016/j.jpsychires.2021.05.037. 34098387

[23] Hammer JH, Vogel DL, Grzanka PR, Kim N, Keum BT, Adams C, et al. The integrated behavioral model of mental health help seeking (IBM-HS): a health services utilization theory of planned behavior for accessing care. J Couns Psychol. 2024;71(5):315–27. 10.1037/cou0000754. 39115906

[24] Hon K, Hamamura T, Hasking P, Lim E, Hon K, Boyes M. Applying social cognitive theory to understanding the disclosure of non-suicidal self-injury: a scoping review. Clin Psychol Rev. 2026;123:102685. 10.1016/j.cpr.2025.102685. 41325707

[25] Simone AC, Hamza CA. Examining the disclosure of nonsuicidal self-injury to informal and formal sources: a review of the literature. Clin Psychol Rev. 2020;82:101907. 10.1016/J.CPR.2020.101907. 32891855

[26] Sheehan L, Nieweglowski K, Corrigan P. The stigma of personality disorders. Curr Psychiatry Rep. 2016;18(1):11. 10.1007/s11920-015-0654-1. 26780206

[27] Tickell A, Fonagy P, Hajdú K, Obradović S, Pilling S. ‘Am I really the priority here?’: help-seeking experiences of university students who self-harmed. BJPsych Open. 2024;10(e40). 10.1192/bjo.2023.652. PMC10897682 38297500

[28] Zanarini MC, Vujanovic AA, Parachini EA, Boulanger JL, Frankenburg FR, Hennen J. A screening measure for BPD: the McLean screening instrument for borderline personality disorder (MSI-BPD). J Pers Disord. 2003;17(6):568–73. 10.1521/pedi.17.6.568.25355. 14744082

[29] Hammer JH, Parent MC, Spiker DA. Mental help seeking attitudes scale (MHSAS): development, reliability, validity, and comparison with the ATSPPH-SF and IASMHS-PO. J Couns Psychol. 2018;65(1):74–85. 10.1037/cou0000248. PMC10460514 29355346

[30] Hanson LLM, Westerlund H, Leineweber C, Rugulies R, Osika W, Theorell T, et al. The symptom checklist-core depression (SCL-CD ¡sub¿6/¡sub¿) scale: psychometric properties of a brief six item scale for the assessment of depression. Scand J Public Health. 2014;42(1):82–88. 10.1177/1403494813500591. 23982463

[31] Spitzer RL, Kroenke K, Williams JBW, Löwe B. A brief measure for assessing generalized anxiety disorder. Archives Intern Med. 2006;166(10):1092. 10.1001/archinte.166.10.1092. 16717171

[32] Kaufman EA, Cundiff JM, Crowell SE. The development, factor structure, and validation of the self-concept and identity measure (SCIM): a self-report assessment of clinical identity disturbance. J Psychopathol Behav. 2015;37(1):122–33. 10.1007/s10862-014-9441-2.

[33] James R, Daukantaité D, Nilsson M. A validation of the Swedish self-concept and identity measure (SCIM) and its association with mental health problems. Heliyon. 2023;9(7):e18151. 10.1016/j.heliyon.2023.e18151. PMC10372226 37519721

[34] StataCorp LLC. 2025. Stata statistical software: release 19. College Station, TX.

[35] Muthén LK, Muthén BO. Mplus User’s Guide. 8th. Los Angeles, CA: Muthén and Muthén; 2017.

[36] Masyn K. Latent class analysis and finite mixture modeling. In: Todd DL, editor. Latent class analysis and finite mixture modeling. Vol. 2. New York: Oxford University Press; 2013. p. 551–611.

[37] Kami M, Pourshahbaz A, Rezaei O, Akhlaghi AAK, Banihashem S, Dehghanizadeh Z, et al. Clinical utility and diagnostic validity of the hierarchical taxonomy of psychopathology for borderline personality disorder. Iran J Psychiatry Behav Sci. 2024;18(4):10.5812/ijpbs–143833. 10.5812/ijpbs-143833.

[38] Meszaros G, Horvath LO, Balazs J. Self-injury and externalizing pathology: a systematic literature review. BMC Psychiatry. 2017;17(1):160. 10.1186/s12888-017-1326-y. PMC5415783 28468644

[39] Edmonds J, Masuda A, Tully EC. Relations among self-concealment, mindfulness, and internalizing problems. Mindfulness. 2014;5(5):497–504. 10.1007/s12671-013-0204-z.

[40] Clement S, Schauman O, Graham T, Maggioni F, Evans-Lacko S, Bezborodovs N, et al. What is the impact of mental health-related stigma on help-seeking? A systematic review of quantitative and qualitative studies. Psychological Med. 2015;45(1):11–27. 10.1017/S0033291714000129. 24569086

[41] Ring D, Lawn S. Stigma perpetuation at the interface of mental health care: a review to compare patient and clinician perspectives of stigma and borderline personality disorder. J Ment Health. 2025;34(1):57–77. 10.1080/09638237.2019.1581337. 30862201

[42] Eaton NR, Krueger RF, South SC, Simms LJ, Clark LA. Borderline personality disorder co-morbidity: relationship to the internalizing–externalizing structure of common mental disorders. Psychological Med. 2011;41(5):1041–50. 10.1017/S0033291710001662. PMC3193799 20836905

[43] Bailey AJ, Finn PR. Borderline personality disorder and the internalizing–externalizing spectrum: a meta-analytic review. Personality Disord: Theory Res Treat. 2019;10(2):143–55. 10.1037/per0000304.

[44] Vogel DL, Wade NG, Haake S. Measuring the self-stigma associated with seeking psychological help. J Couns Psychol. 2006;53(3):325–37. 10.1037/0022-0167.53.3.325.

[45] Kim N, Young CC, Kim BR, Rew L, Westers NJ. Help-seeking behaviors in adolescents and young adults who engage in nonsuicidal self-injury: an integrative review. J Adolesc Health. 2025;77(2):191–210. 10.1016/j.jadohealth.2025.04.015. 40536470

[46] McLaren T, Peter LJ, Tomczyk S, Muehlan H, Schomerus G, Schmidt S. The seeking mental health care model: prediction of help-seeking for depressive symptoms by stigma and mental illness representations. BMC Public Health. 2023;23(1):69. 10.1186/s12889-022-14937-5. PMC9831378 36627597

[47] Peel-Wainwright K, Hartley S, Boland A, Rocca E, Langer S, Taylor PJ. The interpersonal processes of non-suicidal self-injury: a systematic review and meta-synthesis. Phychol Psychotherapy: Theory Res Pract. 2021;94(4):1059–82. 10.1111/papt.12352. 34090311

[48] Mughal F, Troya MI, Dikomitis L, Chew-Graham CA, Corp N, Babatunde OO. Role of the GP in the management of patients with self-harm behaviour: a systematic review. Br J Gen Pract. 2020;70(694):e364–73. 10.3399/bjgp20X708257. PMC7015161 32041771

[49] James R, Lundgren E, Daukantaité D, Nilsson M. From prevalent to personal: how social exposure predicts attitudes toward non-suicidal self-injury and what prevalence reveals. Front Phychol. 2025;16. 10.3389/fpsyg.2025.1652207. PMC12641232 41293103

[50] Trull TJ, Hepp J. Borderline personality disorder: contemporary approaches to conceptualization and etiology. In: Krueger RF, Blaney PH, editors. Oxford textbook of psychopathology. New York: Oxford University Press; 2023. p. 650–77.

