# Supplementary material for: Help-seeking patterns for non-suicidal self-injury across borderline symptom profiles in a large sample of university students
Source: Borderline Personal Disord Emot Dysregul. 2026 Sep 25;13:30. doi: 10.1186/s40479-026-00375-x (PMC13615598; doi:10.1186/s40479-026-00375-x)
Supplement: Supplementary file 1 [file 40479_2026_375_MOESM1_ESM.docx]

**Supplementary Tables**

**Supplementary Table S1. Descriptive comparisons of help‑seekers vs non‑help‑seekers (lifetime NSSI subgroup)**

|  | **NSSI** | | **Difference**  **[95% CI]** | **Effect size (*d*)** |
| --- | --- | --- | --- | --- |
|  | **Help-seeker** | **Non-help-seeker** |  |  |
| **N** | 304 (52.32%) | 277 (47.68%) |  |  |
| **Demographics**  Age, years, M (SD)  Age range, years  Female  Living with others  In a relationship | 24.92 (4.20)  19-76  242 (79.61%)  176 (59.66%)  148 (48.68%) | 25.65 (6.09)  19-44  212 (76.53%)  167 (61.85%)  129 (46.57%) | -0.73 [-1.59, 0.14]  3.07 [-3.67, 9.81]  -2.19 [-10.25, 5.87]  2.11 [-6.01, 10.24] | .14 |
| **Clinical variables,** M (SD)  BPD symptoms (MSI-BPD)  Depression (SCL-CD6)  Anxiety (GAD-7)  Identity disturbance (SCIM)  *Consolidated Identity*  *Disturbed Identity*  *Lack of Identity* | 4.92 (2.79)  18.75 (6.77)  17.32 (5.96)  84.16 (25.61)  47.90 (9.38)  31.24 (11.22)  20.82 (8.88) | 4.32 (2.63)  17.90 (6.66)  16.25 (5.92)  82.10 (26.71)  48.33 (10.19)  31.21 (11.24)  19.23 (8.75) | 0.60 [0.16, 1.05]  0.86 [-0.24, 1.95]  1.07 [0.10, 2.04]  2.06 [-2.21, 6.33]  -0.42 [-2.02, 1.18]  0.04 [-1.79, 1.87]  1.60 [0.16, 3.03] | .22  .13  .18  .08  .04  .00  .18 |
| **Repetitive NSSI (> 5 times)**  Never  Within last 6 months only  More than 6 months ago  Both | 60 (19.74%)  1 (0.33%)  203 (66.78%)  40 (13.16%) | 120 (43.32%)  6 (2.17%)  134 (48.38%)  17 (6.14%) | -23.58 [-30.94, -16.23]  -1.84 [-3.67, -0.01]  18.40 [10.48, 26.32]  7.02 [2.28, 11.76] |  |
| **MHSAS,** M (SD) | 5.41 (1.12) | 5.27 (1.26) | 0.14 [-0.6, 0.33] | .12 |
| **Encouraged to seek help**  Yes | 198 (65.13%) | 80 (28.88%) | 36.25 [28.69, 43.81] |  |
| By formal sources only  By informal sources only  By both sources | 12 (6.15%)  111 (56.92%)  72 (36.92%) | 5 (6.33%)  63 (79.75%)  11 (13.92%) | -0.18 [-6.52, 6.17]  -22.82 [-34.09, -11.56]  23.00 [12.79, 33.20] |  |

*Note.* Difference [95% CI] for continuous variables = mean difference (help-seekers minus non-help-seekers) and use Cohen’s *d* for effect size; for categorical variables = risk difference in percentage points (help-seekers minus non-help-seekers).

MSI-BPD = McLeans Screening Instrument for BPD; SCL-CD6 = Symptom Checklist - Core Depression; GAD7 = Generalised Anxiety Disorder; DERS-16 = Difficulties in Emotion Regulation Scale (Brief Version); SCIM = Self-Concept and Identity Measure. MHSAS = Mental Help-Seeking Attitudes Scale. Formal sources that encouraged them include school staff and healthcare workers, while informal sources that encouraged them include friends, partners, other family members. Formal sources of help sought include primary healthcare, psychiatry, student health, private healthcare, while informal sources of help sought include family and friends, internet/social media, community, and helplines.

**Supplementary Table S2. Latent class model criterion for model selection**

| K | logLik | AIC | aBIC | BIC | entropy | LMR | BLRT |
| --- | --- | --- | --- | --- | --- | --- | --- |
| 2 | -7437.960 | 14913.919 | 14954.207 | 15014.564 | 0.735 | 0.000 | 0.000 |
| 3 | -7318.514 | 14695.027 | 14756.519 | 14848.634 | 0.718 | 0.000 | 0.000 |
| **4** | **-7253.965** | **14585.929** | **14668.624** | **14792.516** | **0.665** | **0.000** | **0.000** |
| 5 | -7234.472 | 14566.945 | 14670.844 | 14826.502 | 0.646 | 0.051 | 0.000 |
| 6 | -7219.869 | 14557.739 | 14682.842 | 14870.267 | 0.684 | 0.219 | 0.030 |

*Note.* Models are derived via maximum likelihood estimation with robust standard errors. logLik = Log Likelihood; AIC = Akaike’s Information Criterion; aBIC = adjusted Bayesian Information Criterion; BIC = Bayesian Information Criterion; LMR = Lo-Mendell-Rubin; BLRT = Bootstrap Likelihood Ratio Test.

**Supplementary Table S3. Help-source denominators and counts by profile among help-seekers (n=297)**

| **Help-sought** | **Full spectrum (n=46)** | **Internalizing (n=84)** | **Externalizing (n=56)** | **Asymptomatic (n=111)** | **Total (n=297)** |
| --- | --- | --- | --- | --- | --- |
| **Formal only** | 15 | 38 | 12 | 40 | 105 |
| **Informal only** | 2 | 6 | 2 | 11 | 21 |
| **Both** | 29 | 40 | 42 | 60 | 171 |

Note. Counts are posterior‑weighted expected counts based on DCAT class probabilities and overall class proportions; rows sum to n help‑seekers. Inference in the main text (Table/Figure) uses three‑step DCAT (posterior‑weighted), which adjusts for classification error. Category mapping: Formal only, Informal only, Both (Cat1–3).

**Supplementary Table S4. Prevalence of key binary predictors by borderline symptom profile among students with lifetime NSSI (n = 581)**

| **Predictor** | **Full spectrum (n=163)** | **Internalizing (n=195)** | **Externalizing (n=95)** | **Asymptomatic (n=128)** | **Total (n=581)** |
| --- | --- | --- | --- | --- | --- |
| **Recent rep. NSSI** | 26 (16.0%) | 27 (13.9%) | 7 (7.4%) | 4 (3.1%) | 64 (11%) |
| **Past rep. NSSI** | 120 (73.6%) | 133 (68.2%) | 65 (68.4%) | 76 (59.4%) | 394 (67.8%) |
| **Encouragement** | 94 (57.7%) | 85 (43.6%) | 44 (46.3%) | 55 (43.0%) | 278 (47.9%) |

Note. Values are most‑likely (modal) class counts from the indicator‑only LCA, with column percentages in parentheses, calculated in the lifetime‑NSSI subgroup (n = 581). Predictors are coded as: Recent repetition = >5 episodes within the last 6 months; Past repetition = >5 episodes more than 6 months ago; Encouragement = ever encouraged to seek help. These counts are provided to contextualize precision in the within‑profile odds ratios in Table 4. Per‑profile denominators in Table 4 come from the one‑step mixture model (not the indicator‑only LCA), so they can differ from the counts reported here.
